# Efficacy of azacitidine is independent of molecular and clinical characteristics - an analysis of 128 patients with myelodysplastic syndromes or acute myeloid leukemia and a review of the literature

**DOI:** 10.18632/oncotarget.25328

**Published:** 2018-06-12

**Authors:** Andrea Kuendgen, Catharina Müller-Thomas, Michael Lauseker, Torsten Haferlach, Petra Urbaniak, Thomas Schroeder, Carolin Brings, Michael Wulfert, Manja Meggendorfer, Barbara Hildebrandt, Beate Betz, Brigitte Royer-Pokora, Norbert Gattermann, Rainer Haas, Ulrich Germing, Katharina S. Götze

**Affiliations:** ^1^ Department of Hematology, Oncology and Clinical Immunology, Heinrich-Heine University, Duesseldorf, Germany; ^2^ Department of Medicine III, Hematology and Oncology, Technische Universität München, Munich, Germany; ^3^ MLL Munich Leukemia Laboratory, Munich, Germany; ^4^ Institute for Medical Informatics and Biometry, Ludwig-Maximilians-Universität, Munich, Germany; ^5^ Institute for Human Genetics, Heinrich-Heine University, Duesseldorf, Germany

**Keywords:** myelodysplastic syndromes, azacitidine, hypomethylating agents, response prediction, targeted therapy

## Abstract

Azacitidine is the first drug to demonstrate a survival benefit for patients with MDS. However, only half of patients respond and almost all patients eventually relapse. Limited and conflicting data are available on predictive factors influencing response. We analyzed 128 patients from two institutions with MDS or AML treated with azacitidine to identify prognostic indicators. Genetic mutations in *ASXL1, RUNX1, DNMT3A, IDH1, IDH2, TET2, TP53, NRAS, KRAS, FLT3, KMT2A-PTD, EZH2, SF3B1,* and *SRSF2* were assessed by next-generation sequencing.

With a median follow up of 5.6 years median survival was 1.3 years with a response rate of 49%. The only variable with significant influence on response was del(20q). All 6 patients responded (*p* = 0.012) but survival was not improved. No other clinical, cytogenetic or molecular marker for response or survival was identified. Interestingly, patients from poor-risk groups as high-risk cytogenetics (55%), t-MDS/AML (54%), TP53 mutated (48%) or relapsed after chemotherapy (60%) showed a high response rate. Factors associated with shorter survival were low platelets, AML vs. MDS, therapy-related disease, *TP53* and *KMT2A*-*PTD*. In multivariate analysis anemia, platelets, *FLT3*-ITD, and therapy-related disease remained in the model. Poor-risk factors such as del(7q)/-7, complex karyotype, *ASXL1, RUNX1, EZH2,* and *TP53* did not show an independent impact. Thus, no clear biomarker for response and survival can be identified. Although a number of publications on predictive markers for response to AZA exist, results are inconsistent and improved response rates did not translate to improved survival. Here, we provide a comprehensive overview comparing the studies published to date.

## INTRODUCTION

Azacitidine (AZA) and decitabine (DAC) are hypomethylating agents (HMAs) commonly used to treat myelodysplastic syndromes (MDS) and acute myeloid leukemia (AML) when not eligible for intensive chemotherapy and allogeneic transplant [1–4]. AZA was the first drug to demonstrate a survival benefit in a randomized trial for patients with MDS. Although HMAs have improved our treatment options for MDS patients considerably, only about half of the patients respond, responses often occur late after several months of therapy and drug treatment remains palliative, leaving allogeneic stem cell transplantation (allo-HSCT) as the only curative option. Transplantation, of course, is only feasible in a relatively small subgroup of patients [5].

To date, limited data is available on factors influencing response to AZA. The French GFM group identified a number of clinical factors relevant for response rate or survival [6]. Not all of these factors, especially regarding response, could be confirmed in subsequent studies [7–10] Regarding cytogenetic or molecular studies several publications exist, but most were retrospective analyses and considerable differences exist regarding subgroups of patients treated, drugs and dosages applied, pretreatment, response criteria as well as laboratory and statistical methods, making interpretation of results and comparisons between different studies difficult [7, 8, 10–13]. Cytogenetics were only partly addressed and no study to date concentrated on specific chromosomal aberrations. In addition, many previous studies analyzed only one marker or combined analyses of patients treated with azacitidine and decitabine, although it has not been clearly shown that both drugs act in exactly the same way [1, 14, 15].

Due to the number of mutations found in epigenetic regulators, it has been hypothesized that such mutations may impact response to AZA and indeed, an early publication suggested the utility of *TET2* mutations as a predictive marker for AZA response [16] which was partly confirmed by a second group [8]. However, both studies showed an almost identical survival for patients with and without the mutation and others could not confirm the value of *TET2* for response prediction [7, 10, 17]. *TP53* mutations, despite high response rates to HMAs [18, 19], have emerged as a clear marker for poor survival, even when allogeneic transplantation is performed [20, 21]. Retrospective data on superior response of *DNMT3A*-mutated patients to HMAs have been reported, but were only described for AML [11, 22]. In summary, no unambiguous evidence for a molecular marker predicting response to HMAs has been uncovered to date. The present study is one of the largest on patients treated with AZA. We provide a detailed analysis of relevant prognostic factors on response and survival in AZA treated patients including clinical variables, conventional cytogenetics as well as the most frequent molecular aberrations. With the exception of 11 patients treated for severe cytopenias, all patients belonged to the intermediate and high risk category in accordance with the AZA001 study population.

## MATERIALS AND METHODS

### Patients and treatment

The study population included 128 patients treated between 8/2004 and 12/2014. Patients were treated at two institutions, either the Heinrich-Heine University Hospital in Düsseldorf or at the Technische Universität München in Munich. AZA was applied either at the approved schedule (75 mg/m^2^/d for 7 days), as a 5-2-2 regimen with a weekend break or as a five-day regimen with increased dosage (100 mg/m^2^/d for 5 days). To be evaluable, all patients had to have had at least 3 cycles of azacitidine. Treatment consisted of AZA monotherapy or, in a few cases, a combination with valproic acid [23]. Diagnoses of MDS, MDS/MPD, and AML (either as sAML after MDS or AML with myelodysplasia-related changes) were included. Patients who had undergone allogeneic HSCT before AZA treatment were excluded from the study, while in 22 (17%) patients allogeneic HSCT followed AZA treatment. All samples were collected with written informed consent in accordance with the declaration of Helsinki.

### Definitions of response and survival

Response to AZA treatment was assessed using the modified international working group criteria (IWG 2006) [24]. Patients achieving complete remission (CR), partial remission (PR), marrow CR with hematological improvement (mCR with HI), or stable disease with HI (SD with HI) were considered as responders. Survival was defined from start of treatment until death. Patients were censored at the time of last observation, when still alive.

### Gene mutation analysis

All patients were analyzed by next-generation-sequencing using a myeloid gene panel containing *ASXL1, RUNX1, DNMT3A, IDH1, IDH2, TET2, TP53, NRAS, KRAS, EZH2, SF3B1* and *SRSF2* as described previously [25, 26]. Briefly, libraries of 12 genes were generated either with the ThunderStorm (RainDance Technologies, Billerica, MA) or the Access Array System (Fluidigm, San Francisco, CA). Libraries were sequenced and demultiplexed on a MiSeq instrument (Illumina, San Diego, CA). The FASTQ files were further processed using the Sequence Pilot software version 4.1.1 Build 510 (JSI Medical Systems, Ettenheim, Germany) for alignment and variant calling. Variant allele frequency for variant calling was set to a limit of ≥3%. *KMT2A-PTD* was analyzed by real-time PCR described in detail previously [27]. *FLT3*-ITD was analyzed by gene scan as described previously [28].

### Statistical methods

Response probabilities were analyzed using Fisher´s exact test or logistic regression models, as applicable. Survival probabilities were analyzed using Kaplan-Meyer curves and proportional hazards models. *P* values < 0.05 were considered significant. Due to the exploratory character of this work, all *p* values were interpreted descriptively, no adjustment was done.

## RESULTS

### Patient characteristics

A total of 128 patients treated with AZA in a 10-year period between 2004 and 2014 were included. Median age was 70 (49–84) years. According to WHO 2016 [29] 51 (40%) patients had AML, 68 (53%) had MDS, and 9 (7%) MDS/MPD, while according to FAB criteria the distribution was 25% AML and 75% MDS. The majority of MDS patients had an IPSS score of INT-2 or High [30]. Only 9% of patients (*n =* 9) belonged to the INT-1 risk group, all with either increased blast counts or high-risk cytogenetics, while only 2 patients (2%) belonged to the low-risk group, but had extremely severe cytopenias. In line with this, none of the patients belonged to the very low-risk IPSS-R group, while only 5 (5%) were categorized as low-risk. Regarding cytogenetics 69 (54%) had a good-risk karyotype, 21 (16%) an intermediate-, and 38 (30%) a poor-risk karyotype according to IPSS-R [31]. On average 2.2 molecular abnormalities were identified per patient. The most frequent mutations were *ASXL1* (35%), *SRSF2* (34%), and *RUNX1* (28%). 89% of patients had mutations in at least 1 of the investigated genes. A substantial percentage of patients were therapy-related (19%). With regard to pretreatment, 10 (8%) patients had previously received an intensive chemotherapeutic regimen, 33 received other, non-intensive drugs (i.e. erythropoeitin, valproic acid, low dose Ara-C, investigational drugs), and 85 (66%) patients were treatment-naïve. Patient characteristics are summarized in Supplementary Table 1.

### Treatment modalities and response

Median duration of MDS before onset of therapy was 185 days. Median follow-up was 5.6 years. Patients survived for a median of 1.3 years (1.2 years for AML patients only and 1.5 years for MDS IPSS INT-2/High). The median number of given cycles was 6 (3–58). 116 patients received AZA monotherapy (91%), while 12 patients received AZA plus valproic acid within a clinical trial (9%). Response rates were slightly higher in MDS (52%) as opposed to AML patients (47%) (all patients: 49%). Regarding the quality of responses 15 (12%) of responding patients achieved CR, 6 (5%) marrow CR (mCR) with HI, 15 (12%) PR, and 28 (22%) SD with HI. Of the non-responders 3% each had mCR or PR without HI, 36 (28%) had SD without HI, and 20 (16%) had PD. Response characteristics and survival are shown in Supplementary Table 2.

### Predictive factors for response–clinical features, classification, and cytogenetics

To determine baseline characteristics that might predict response to AZA we first performed univariate analyses. No clinical parameter including morphologic and other prognostic variables was significantly associated with response to AZA. A weak association with an inferior response was observed for the cytogenetic intermediate-risk group compared to the poor-risk group (29 vs 55%, *p* = 0.053), while the response rate was 52% in patients with good-risk karyotype. Response rates were highest in patients with CMML (6/8), and 6 out off 7 patients with CMML belonging to the IPSS INT-2/High category, patients with RA according to FAB (4/4), IPSS-R intermediate (7/10), and IPSS-R very low-risk cytogenetic group (3/4), although subgroups were small. Patients with poor risk features such as high-risk cytogenetics (55%) according to IPSS, very poor-risk cytogenetics according to IPSS-R (55%), t-MDS/AML (54%), or previous intensive chemotherapy (60%) exhibited surprisingly good response rates even slightly above average.

Regarding cytogenetics, the most interesting observation was a response in 6 out off 6 patients with del(20q), *p* = 0.012. For other cytogenetic subtypes the response rate was slightly lower (40%) in patients with del(5q) while 3 out off 4 patients with –Y responded. As could be assumed from the good results for the poor/very poor cytogenetic risk groups (IPSS/IPSS-R), patients with chromosome 7 abnormalities (52%) or complex karyotypes (52%) also responded well.

### Predictive factors for response–molecular mutations

We next looked at molecular abnormalities and found that similar to cytogenetics no single molecular mutation was predictive of response to AZA. Response rates were highest in patients with *IDH2* (63%) and *NRAS* mutations (62%), while they were low in patients with *KMT2A-PTD* (1/4), *IDH1* (3/10), *FLT3*-ITD (3/10), and *EZH2* mutations (3/10), but none of these differences reached statistical significance. Considering only patients with a certain VAF (≥10%) did not impact our results. We then looked at the most frequent marker combinations, namely *RUNX1* + *TET2* (*n =* 11), *SRSF2* + *TET2* (*n =* 15), *ASXL1* + *TET2* (*n =* 11), *SRSF2* + *ASXL1* (*n =* 23), *SRSF2* + *RUNX1* (*n =* 18), *ASXL1* + *RUNX1* (*n =* 18), *IDH2* + *ASXL1* (*n =* 9), and *EZH2* + *ASXL1* (*n =* 7), and *RUNX1* + *ASXL1* + *SRSF2* (*n =* 10). No influence on response was observed for any of the above mentioned groups and this was also the case for the most frequent combinations of certain molecular mutations with cytogenetic abnormalities (i.e. *DNMT3A, RUNX1, SRSF2*, and *TET2* within a normal karyotype; *TP53* in patients with complex karyotype or del(5q); 5q- or 7q- within a complex karyotype and combinations of chromosome 7 and 5 abnormalities). Finally, we grouped genes into epigenetic (together or separated as histone modifying (*EZH2*, *ASXL1*, *KMT2A-PTD*) and methylation relevant (*TET2*, *DNMT3A*, *IDH1/2*), splicing factor (*SF3B1*, *SRSF2*), and proliferation enhancing (*NRAS*, *KRAS*) mutations. The highest response rate was seen for patients with *N*- and *KRAS* mutations (60% all patients, 73% MDS IPSS int2/high). The lowest response rate was observed in patients with splicing factor mutations (39, and 35% respectively; *p* = 0.066, see Supplementary Table 2). With increasing number of mutations there was a decrease in response rates, but this difference did not reach statistical significance (62%, 55%, 46%, and 43% for 0, 1, 2–3, and >3 mutations present, respectively).

To minimize influences of inhomogeneous patient populations we performed all analyses for response and survival in a subgroup limited to intermediate- and high-risk MDS (IPSS) as well as in patients receiving AZA monotherapy. A part of the analysis was also performed in AML patients only, but the informative value was limited due to small patient numbers. No relevant differences were observed (Table 1, Supplementary Table 2).

**Table 1:** Multivariable analysis for survival

Variable	HR	95%-KI	*P*
Anemia - 1 vs. 0	2.32	1.36; 3.95	0.002
1.5 vs. 0	1.52	0.74; 3.11	0.255
Thrombocytopenia - 0.5 vs. 0	0.92	0.49; 1.72	0.787
1 vs. 0	1.90	1.08; 3.34	0.027
*IDH1*	2.37	1.05; 5.39	0.040
*TP53*	2.02	1.15; 3.53	0.014
*FLT3*- ITD	2.87	1.28; 6.42	0.010
*KMT2A-PTD*	2.67	0.91; 7.88	0.075
Subtype of disease–primary vs. secondary	2.86	1.61; 5.07	<0.001

### Influence of different definitions for response

To assess if or how different definitions of response may influence the results, we performed our analyses with 3 further definitions of response: response 2 = CR, PR, and mCR with HI; response 3 = CR, PR, mCR with HI, mCR without HI; response 4 = CR, PR, mCR with HI, mCR without HI, SD with HI. The highest CR/PR rates (response 2) were seen for patients with del20q (3/3), minus Y (2/4), chromosome 7 abnormalities (8/23), complex karyotypes (7/21), *KRAS* (2/5), *DNMT3A* (7/21), *TP53* (8/25) and *NRAS* (4/13) mutations. The lowest CR/PR rates were seen for *EZH2* (1/10), *FLT3*-ITD (1/10), *SRSF2* (4/39; p = 0.002), and *TET2* mutations (3/28; *p* = 0.029). With inclusion of mCR without HI the highest CR/PR rates were seen for del20q (3/3), −Y (2/4), +8 (8/17), KRAS (3/5), and *IDH1* mutations (4/10) and the lowest for EZH2 (1/10), FLT3 (1/10), TET2 (4/28; *p* = 0.013), and *SF3B1* mutations (2/11). In this model anemia (Hb 8–10 g/dl) was an additional factor for a lower response rate (*p* = 0.034). Finally, with our broadest response definition patients with del(20q) (6/6; *p* = 0.032), −Y (3/4), +8 (12/17), *IDH2* (12/16), *NRAS* (9/13), and *KRAS* mutations (3/5) showed the best response rates, patients with *KMT2A-PTD* (1/4), *FLT3*-ITD (3/10), and *EZH2* mutations (3/10) the worst. As with our primary definition, no other variable except del(20q) significantly influenced response.

### Predictive factors for survival

Among clinical variables platelet count <50.000/µl was associated with shorter survival (HR 1.78) while a Hb value < 10 g/dl was only significant in the IPSS int2/high subgroup (HR 1.94). Patients with MDS (vs. AML; according to WHO (HR 0.56) as well as FAB (HR 0.56)) and patients with de-novo (vs. therapy-related) disease (HR 1.99) fared better. Furthermore, patients with RAEB according to FAB (*n =* 63) (HR 0.57), CMML (*n =* 8, HR 0.45), and RA (*n =* 8, HR 0.42) lived longer than patients with AML according to FAB, although this difference achieved statistical significance only for RAEB, possibly due to different sample sizes. The same was true for lower-risk IPSS (*n =* 11, HR 0.18) and IPSS-R (*n =* 5, HR 0.43) categories. Interestingly, patients with lower-risk cytogenetics according to IPSS and IPSS-R lived longer when compared to the higher-risk groups (HR 0.74), although this difference was not statistically significant.

Although associated with the highest response rate, deletion 20q was not associated with improved survival (HR 1.07), while the presence of a complex karyotype only significantly influenced survival in the MDS INT2/High-risk subgroup (1.89; all patients HR 1.68). Regarding molecular abnormalities, only patients with *TP53* mutations (HR 1.80) and *KMT2A-PTD* (HR 3.39) had a significantly shorter survival, but the latter group was small (*n =* 4). The effect of small numbers can be demonstrated in patients with *EZH2* mutations, since this mutation had a dismal influence in the MDS INT2/High subgroup (*n =* 4; HR 8.55; *p* = 0.001), while the influence of the same mutation was favorable in AML patients (*n =* 4; HR 0.09; *p* = 0.022)

**Figure 1:**
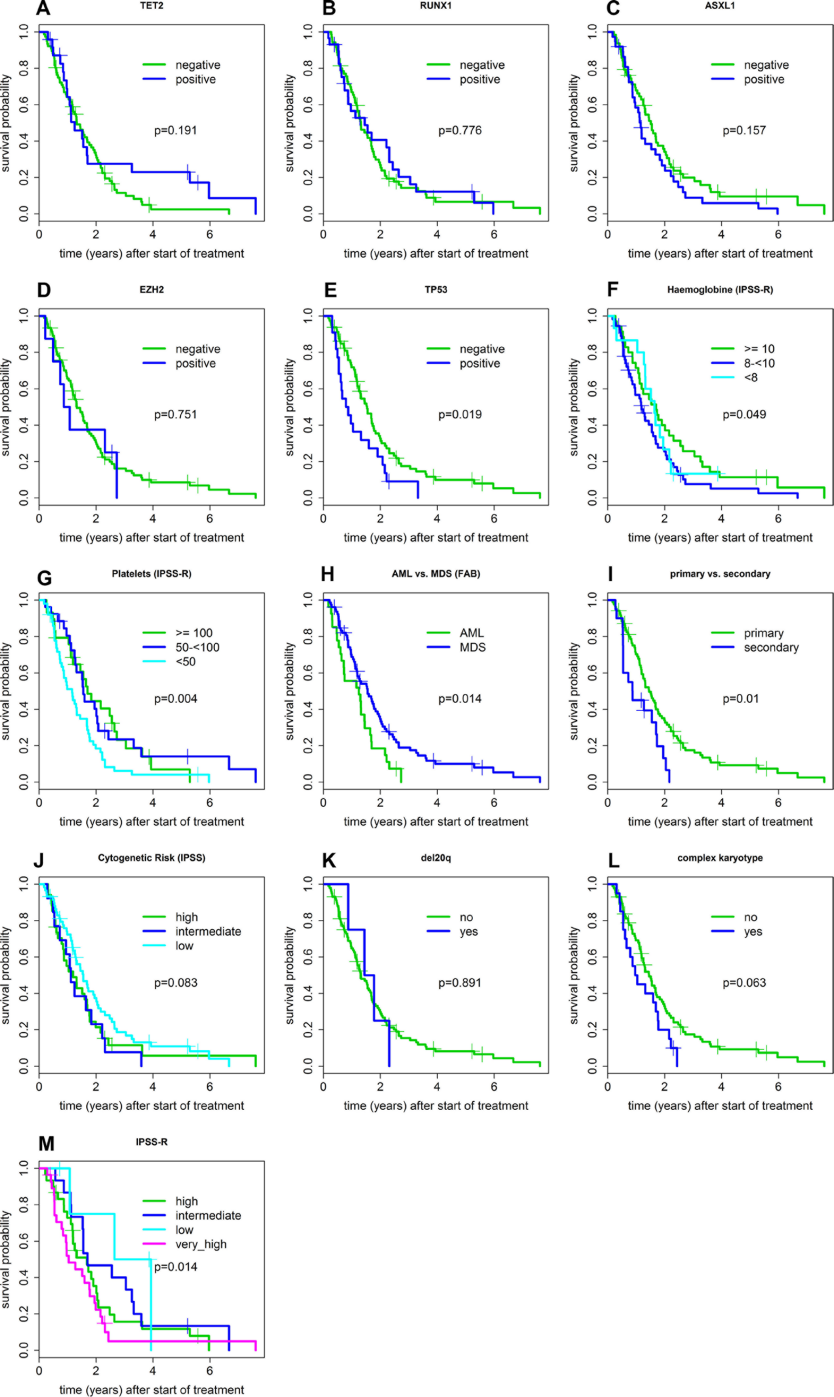
Prognostic variables influencing survival probability of patients treated with AZA

Of note, patients with *TET2* mutations did not show an improved survival compared to *TET2* wild-type patients (HR 0.71). Remarkably however, we observed a long median survival for patients with a combination of *TET2* and *RUNX1* mutations, namely 3.3 years for all patients (*n =* 11; HR 0.46; *p* = 0.068) and 5.3 years in MDS INT2/High (*n =* 6; HR 0.30; *p* = 0.049), longer than all other cytogenetic or molecular subgroups. All other marker combinations, except *TP53* plus complex karyotype, did not significantly influence overall survival (HR 2.02; *p* = 0.015 see Figure 1). All factors for survival are shown in Supplementary Table 2.

### Multivariable analyses

We attempted to define a multivariate model for response. However, due to restricted patient sample size and the strong correlations between covariates, results depended heavily on the variable selection procedures chosen. Therefore, no stable model could be achieved.

Multivariable analysis for survival was conducted taking into account the following variables with a *p*-value of < 0.1 in univariable analysis: *TP53*, *IDH1*, *FLT3*-ITD, *KMT2A-PTD*, complex karyotype, anemia (Hb 8–10 g/dl), thrombocytopenia (platelets <50.000/µl), AML vs. MDS (FAB), cytogenetic risk group (IPSS), primary vs. secondary MDS. The analysis was stratified for age and gender. Interestingly, *TP53* mutations as well as complex karyotype did not independently influence survival. Anemia (Hb 8–10 g/dl), platelets <50.000/µl, *FLT3*-ITD, and subtype of AML (primary vs. secondary) were the variables with a significant influence on survival in our model. Multivariable analysis is shown in Table 1.

## DISCUSSION AND REVIEW OF THE LITERATURE REGARDING RESPONSE PREDICTORS FOR AZACITIDINE TREATMENT

### Summary and discussion of results

In this substantial comprehensive analysis on response characteristics and survival in 128 patients treated with AZA, we could not identify a clear response predictor to treatment. Although certainly not as large a cohort as has been described for untreated MDS patients [32], our cohort is one of only two larger reports to date for patients treated homogenously with AZA [9], while most others have combined patients who received AZA or DAC [7, 8] (Supplementary Table 3).

It was an interesting finding that patients with del(20q) responded in all cases although this finding will need confirmation in a larger cohort. We could not confirm previously published improved response rates for patients with *TET2* mutations. Importantly, our results were independent of clone size or marker combinations analyzed.

A unique feature of our analysis is the minimum number of 3 administered AZA cycles. This excludes patients who already had progressive disease at start of treatment or were to unfit due to comorbidities or infection, received only 1–2 cycles and might not have received enough treatment to respond. Response rates and median numbers of cycles given compare very well to previous publications and suggest our patient cohort is representative [8, 9].

We observed a very good response rate in high-risk subgroups (i.e. chromosome 7 abnormalities, complex karyotype, high-risk cytogenetics, *TP53*, previous chemotherapy, and therapy-related disease). We and others have previously reported a high response rate for *TP53* mutated cases and a favorable response rate has also been observed for MDS with monosomal karyotype treated with decitabine [18, 19, 33]. In the multivariate analysis, only primary vs. secondary MDS stayed in the model. Furthermore, patients with poor-risk molecular subtypes such as *ASXL1*, *EZH2*, *NRAS*, or *RUNX1* did not exhibit a survival disadvantage. This might in part be due to sample size and strong correlation between variables. However, this seems especially obvious when comparing hazard ratios in patients with *RUNX1* (HR 0,67 in our study (subgroup *RUNX* plus *TET2* 0,30) vs. 2,82 in Haferlach *et al.*, Leukemia 2014) and *NRAS* mutations (HR 0,87 vs. 3,07), who live even slightly longer while untreated patients exhibit a significantly inferior survival [34, 35]. Such patients might especially benefit from AZA treatment and these results are in line with the literature [7–9, 35]. However, patients with inferior survival like TP53 mutated, complex karyotypes, or therapy-related MDS still benefit as well. Although their responses might be short-lived, it is not possible from our retrospective data to conclude that they would not show improved survival in a randomized trial since survival of these patients without treatment is extremely short. Comparing hazard ratios for TP53 and complex KT from our cohort with published datasets suggests survival might be better in treated patients (HR 2,14 in our study vs. 4,27 in [34]; HR 1,55 vs 3,8 for the very poor risk IPSS-R group in [30] and HR 4,2 in [31]). We also observed a decline in response to AZA with increasing number of genetic mutations. This corresponds to previously published data from large cohorts demonstrating that prognosis of untreated MDS patients is inversely associated with the number of detected mutations [32, 34]. On the one hand this demonstrates that an influence on response rate exists, since this effect is very likely to become relevant in a larger cohort. On the other hand, as scientifically interesting as this observation may be, it also demonstrates the poor risk group of patients with more than 3 mutations still benefits in >40% of cases.

### Review of prognostic and predictive markers for treatment with HMAs

#### Non-molecular prognostic markers

A number of retrospective studies with relatively inhomogeneous cohorts exists thereby complicating comparisons between trials (Supplementary Table 3). A large analysis of the influence of clinical parameters on response to AZA has been performed by Itzykson *et al.* [6]. While some of the relevant parameters like pretreatment with low-dose Ara-C did not occur in our cohort, others like blast count and abnormal karyotype were not relevant in our group of patients. This absence of clinically relevant predictive/prognostic markers is in line with an analysis from the original AZA001 publication [2] showing a benefit of AZA versus conventional care for all subgroups independent of variables analyzed. A comparable analysis was performed more recently in the AZA-AML-001 study [36].

### Influence of cytogenetic abnormalities

In contrast to analyses of molecular markers, very limited data exists on cytogenetics and AZA response. In a subgroup analysis from AZA001 [37] patients with chromosome 7 abnormalities within a complex karyotype did not show the same survival benefit, compared to patients with isolated chromosome 5 and 7 abnormalities, trisomy 8 isolated or complex, or patients with normal karyotype. However, this analysis focused on survival, not response. Papers on molecular markers have looked at cytogenetic risk groups, but not individual abnormalities. Thus, we cannot compare our findings regarding the high response rate of patients with del (20q), since no other group reported response of cytogenetic subtypes in detail, with the exception of chromosome 7 abnormalities that have been linked to response to AZA as well as decitabine [33, 38, 39].

### Influence of molecular markers-*TET2*

While an early study by the French GFM group has suggested an impact of *TET2* mutations on outcome of AZA treatment [16], the same study group could not find such an effect in CMML patients treated with decitabine [17]. Likewise, studies by other investigators did not find an effect of *TET2* or any other somatic mutation analyzed individually in univariate analyses [7, 8, 10, 11, 40, 41]. Because many *TET2* mutations were subclonal and the French paper had used less sensitive Sanger sequencing, Bejar *et al.* reanalyzed their data by excluding clones with a size of <10% and found a minor improvement in response rate (60 vs. 43%; *p* = 0,04) [8]. This difference was greater if only patients with *TET2* mutations and *ASXL1* wildtype were taken into consideration (74 vs. 44%, *p* = 0,009), although *ASXL1* alone did not affect response or survival. More importantly, survival was not affected in either study, although responders to HMA treatment generally exhibit a prolonged survival compared to non-responders and the presence of a *TET2* mutation itself does not unfavorably affect survival [34, 42]. Thus, the clinical relevance of *TET2* mutations remains questionable.

### Influence of molecular markers: *DNMT3A* and other possible biomarkers

While other papers focused on TET2, Traina and coworkers analyzed a larger cohort of patients (*n =* 92) and a number of different molecular markers [7]. In univariate analysis no marker by itself had significant influence on response rate. If *TET2*, *IDH1/2*, and *DNMT3A* were taken together, a trend was seen for improved response (*p* = 0.06). The combination of *TET2* and *DNMT3A* achieved a small influence in multivariable analysis (*p* = 0.03). This paper cannot be compared well to our results for several reasons. First, AZA (*n =* 55) or DAC (*n =* 26), or both (*n =* 11) were used. Some patients received a combination with lenalidomide. Second, high-risk as well as low-risk patients according to IPSS were included. And third, the response rate was unexpectedly low (24%). Of the 30 higher risk patients only 5 responded making statistical conclusions for this subgroup difficult. In the above mentioned study by Bejar *et al.* a large panel of 40 frequently mutated genes was analyzed in the largest cohort to date (*n =* 213). AZA alone was only received by 42 patients, while the others received DAC or DAC plus another agent [8]. The only clinical feature significantly associated with response was FAB classification, due to a high response rate of CMML patients (81%). No mutation was found to predict response to AZA in univariate analysis. Only when the analysis was repeated on patients with larger clone size, a better response rate was seen for patients with TET2 mutations. Survival data was only available in 146 patients. Mutations negatively affecting survival were *TP53* and *PTPN11*. Two analyses limited to CMML patients evaluated the predictive/prognostic impact of a limited number of genes for HMA response, including *TET2*. None of the genes analyzed showed a significant influence [17]. Other studies with limited number of patients and mixed cohorts were performed using different gene panels. In one study SETBP1 mutations and U2AF1 were predictive for response and non-response, respectively, but the limited number of patients makes it difficult to draw definite conclusions [10, 12, 43]. An analysis on 134 AZA treated higher risk MDS patients evaluated the impact of a large panel of genes on treatment outcome. No mutation was associated with response or survival, but mutations in histone modulators (*ASXL1/EZH2*) led to prolonged survival, while mutations in methylation affecting genes (*TET2*/*DNMT3A*/*IDH1/2*) did not [9].

Mutations in *DNMT3A* and *IDH1/2* were the focus of several other analyses. DiNardo *et al.* evaluated the impact of *DNMT3A* and *IDH1/2* mutations in 68 AML patients treated with AZA, DAC, or combination regimen [13]. No correlation was found. In contrast Metzeler and coworkers found *DNMT3A* mutations to be predictive for response to DAC or DAC in combination with vorinostat in patients with elderly AML [11]. This finding could neither be reproduced in our cohort nor in other studies [7–10, 12] and survival was even inferior in one of these studies [12]. Finally, a recent paper described 83 AML patients treated with hypomethylating agents, including NGS of *DNMT3A*, *IDH1/2*, *TET2* [22]. The authors first analyzed bi-centric data and then performed a combined analysis of 152–239 patients (depending on the mutation) from different publications. In both analyses none of the mutations analyzed impacted CR rate. Concentrating on first-line patients led to an increased response rate for *DNMT3A* mutated patients, especially in *NPM1* co-mutated patients. In our study we did not analyze *NPM1*, since in the first 40 patients sequenced we did not observe a single *NPM1* mutation. Looking at pretreatment, there were no differences regarding response of *DNMT3A* mutated first or later line patients.

### Influence of molecular markers-*TP53*

Several papers analyzed the influence of *TP53* mutations on outcome to HMAs. In all publications response rate is relatively high around 50%, while survival is generally shorter compared to other MDS subtypes [18, 19, 44]. Thus, *TP53* confers an adverse prognosis but is not suitable as a predictor of response. A detailed comparison of these studies can be found in Supplementary Table 3.

### Other clinical and preclinical results on biomarkers for azacitidine response

Supporting our results of no genetic markers predictive for response to AZA a comparative analysis of AZA-sensitive and AZA-resistant SKM1 cell lines showed that, despite differences in gene expression patterns, both cell lines harbored the same mutations [45]. This is further backed up by results from Merlevede and coworkers, which have shown just recently that the mutation allele burden remains unchanged in CMML patients responding to hypomethylating agents [46]. Meldi *et al.* have shown that differentially methylated non-promoter regions of DNA at baseline distinguished responders from non-responders to decitabine, while somatic mutations on the other hand did not [47]. Furthermore, there are other markers that might be relevant including expression of CMYB or CJUN [17], certain cytokines like CXCL4 and CXCL7 or expression of metabolic enzymes and nucleoside transporters needed for their activation as cytidine and deoxycytidine kinase as well as cytidine deaminase [47–50]. It might well be that the effects of HMAs can, at least partially, be ascribed to increased cancer immunogenicity via increased expression of transposable elements and multiple other immune regulatory effects [51–67].

### Comparability and differences of studies on azacitidine response

It is impossible to compare the different studies on predictive markers for therapy with demethylating agents (see Supplementary Table 3) as some of them combined low- and high-risk MDS, included patients with AML or were limited to CMML [17, 68]. In addition, patients who received different hypomethylating agents are mixed in many studies [7, 8, 10, 13, 68]. However, DAC is incorporated into DNA, while AZA is mainly incorporated into RNA and clinical as well as preclinical studies suggest differences between the two drugs [2, 15, 33, 69–72]. In addition, some studies included patients receiving combination regimens [7, 8, 11, 13, 22, 41, 73]. Finally, differing definitions of response are used. Some look at CR and PR only, while others, like us, define response as CR, PR, and HI according to IWG criteria, and others again include marrow remissions without HI. This is, for example, a difference between the study of the French GFM group and our analysis [16]. In the aforementioned study many patients in the *TET2*-mutated group showed marrow remissions. IWG criteria suggest giving information about hematologic improvement in addition to marrow remission but in practice this is often not done. However, studies have shown a comparable survival for patients achieving CR, PR, or HI, suggesting the survival benefit might be linked to the improvement in cell counts rather than reductions in marrow blasts only [2].

Retrospective analyses are difficult and associated with several sources of error. As other patient cohorts were inhomogeneous our cohort is a “real life cohort” as well, including all patients with available material treated at our two institutions. Since our study included patients receiving combination therapy and a few lower risk patients as well, we decided to do all analyses in Int2 or high-risk MDS patients and patients receiving AZA monotherapy separately, in order to minimize the influence of inhomogeneous cohorts. We also performed our analyses in patients with a VAF of ≥10%, but found no differences as compared to the main analysis in any of these subgroups. In our cohort the use of different response definitions led to only minor differences in results.

We have grouped our mutations according to biological mechanisms in order to compare our results to published data. However, it remains uncertain whether this is a good approach. Mutations in DNA methylation and histone regulators are known to be not mutually exclusive, and many combinations exist, suggesting the epigenomic machinery has to be seen as a complicated process [74]. Therefore it is uncertain whether it makes sense to group certain mutations together. A predominantly hypomethylation profile was found in patients with *DNMT3A* mutations, while in contrast *TET2* mutations were associated with a hypermethylation profile, *ASXL1* mutations with hypo- as well as hypermethylated regions, while a spliceosome mutation (*SRSF2*) was linked to the strongest methylation differences [47]. Regarding histone regulators, *ASXL1* encodes a polycomb group protein involved in transcriptional regulation, while *EZH2* encodes for a histone-lysine N-methyltransferase. And finally, spliceosome mutations result in completely different phenotypes as *SF3B1* mutations are predominantly associated with ringed sideroblasts and *SRSF2* with CMML.

## CONCLUSIONS

To conclude, the existing studies on molecular predictive/prospective markers in patients treated with HMAs have yielded disappointing results. From a statistical point of view they all are relatively small. This is especially pertinent if one would like to analyze DAC and AZA treated patients separately or to compare them. In most cases the results found in one study could not be reproduced in the next. This is, despite limited patient numbers, likely due to the fact that HMAs are not targeted therapies in MDS comparable for example to lenalidomide [75, 76]. For AZA, in a large enough subgroup it might be expected that one or the other molecular group responds slightly better or less well and larger patient cohorts to detect more subtle differences are definitely needed and scientifically interesting. But if AZA would act as a molecularly targeted agent, results would have been clearer. None of the findings regarding the predictive value of molecular markers is likely to lead to clinical consequences. Methylation profiles of non-promoter regions, if confirmed in larger patient cohorts might prove to be more reliable tools for response prediction [47]. On the other hand, the multiple different effects of HMAs on immune regulatory mechanisms might play a more important role for their clinical effects as previously appreciated and in contrast to an impact targeted directly against the effects of certain molecular aberrations [51–67]. Currently, none of the response rates are high or low enough to choose certain patients preferentially or to keep others from being treated and effects on survival are missing.

## SUPPLEMENTARY MATERIALS TABLES








