# Supplementary material for: Efficacy of azacitidine is independent of molecular and clinical characteristics - an analysis of 128 patients with myelodysplastic syndromes or acute myeloid leukemia and a review of the literature
Source: Oncotarget. 2018 Jun 12;9(45):27882–94. doi: 10.18632/oncotarget.25328 (PMC6021252; doi:10.18632/oncotarget.25328)
Supplement: Supplementary file 1 [file oncotarget-09-27882-s001.pdf]

Efficacy of azacitidine is independent of molecular and clinical characteristics - an analysis of 128 patients with myelodysplastic syndromes or acute myeloid leukemia and a review of the literature

SUPPLEMENTARY MATERIALS

Supplementary Table 1: Patient characteristics. See Supplementary_Table_1

Supplementary Table 2: Predictive factors for response to AZA and prognostic variables for survival of AZA treated patients. See Supplementary_Table_2

Supplementary Table 3: Comparison of publications on predictive factors for AZA treatment. See Supplementary_Table_3
